# Supplementary material for: How to Catch the Ball: Fullerene Binding to the Corannulene Pincer
Source: Molecules. 2022 Jun 15;27(12):3838. doi: 10.3390/molecules27123838 (PMC9228874; doi:10.3390/molecules27123838)
Supplement: Supplementary file 1 [file molecules-27-03838-s001.zip › CatchTheBall_SI.pdf]

How to Catch the Ball: Fullerene binding to the Corannulene Pincer - Supporting Information

Author: Filipe Menezes; Grzegorz Maria Popowicz

More Detailed Thermodynamic Data on Binding Fullerene to Buckycatcher

Table S1: Most relevant thermodynamic data for the formation of buckycatcher-fullerene complexes according to several semi-empirical methods in gas and with several solvation models. This is all conformer specific data. Gibbs free energies given in $kcal/mol$, whereas entropies are in $cal.K^{-1}.mol^{-1}$.

	$C_{60}@ii$	$C_{60}@ie$	$C_{60}@ee$	$C_{70}@ii$	$C_{70}@ie$	$C_{70}@ee$
ΔH_{gas}^{GFN2}	−39.145	−30.819	−24.790	−40.960	−33.675	−26.533
$\Delta H_{gas}^{PM6-D3H4X}$	−27.394	−21.679	−15.741	−29.307	−23.576	−17.323
$\Delta H_{gas}^{PM6-D3H+}$	−30.217	−23.680	−17.132	−32.203	−25.538	−18.783
ΔS_{gas}^{GFN2}	−47.337	−47.978	−48.765	−50.887	−49.692	−48.496
$\Delta S_{gas}^{PM6-D3H4X}$	−48.212	−45.733	−47.354	−50.424	−49.274	−49.472
$\Delta S_{gas}^{PM6-D3H+}$	−49.670	−46.437	−47.371	−52.533	−49.991	−50.617
ΔG_{gas}^{GFN2}	−24.944	−16.425	−10.161	−25.694	−18.767	−11.984
$\Delta G_{gas}^{PM6-D3H4X}$	−12.931	−7.959	−1.535	−14.179	−8.793	−2.481
$\Delta G_{gas}^{PM6-D3H+}$	−15.316	−9.749	−2.921	−16.443	−10.540	−3.598
$\Delta G_{PhMe}^{GFN2/ALPB}$	−14.309	−8.944	−6.027	−13.417	−10.609	−7.039
$\Delta G_{PhMe}^{PM6-D3H4X/COSMO}$	−10.886	−5.821	0.855	−11.669	−6.415	0.090
$\Delta G_{PhMe}^{PM6-D3H+/COSMO}$	−13.272	−7.611	−0.532	−13.933	−8.162	−1.027
$\Delta G_{PhMe}^{GFN2/COSMO-RS}$	−18.444	—	—	−18.394	—	—
$\Delta G_{PhMe}^{PM6-D3H4X/COSMO-RS}$	−6.431	—	—	−6.879	—	—
$\Delta G_{PhMe}^{PM6-D3H+/COSMO-RS}$	−8.816	—	—	−9.143	—	—

Evaluation COSMO-RS Errors

In order to estimate errors from using the same COSMO-RS solvation energy for all conformers we evaluate the respective quantities using the other two models. The COSMO solvation contributions for the buckycatcher's conformers are -10.834, -10.820 and -10.994 $kcal/mol$ for respectively ii, ie and ee. Though the magnitude of solvation at the ALPB level is larger, differences between conformers are also minimal: -38.164 $kcal/mol$ for ii, -38.148 for ie and -38.145 for ee. In order to evaluate the differences arising from using different geometries for the same conformer we calculated the ALPB solvation contribution for ii in GFN2-xTB's against PM6-D3H4X's optimized geometry. The difference was of 0.727 $kcal/mol$. These are all absolute deviations.

Dynamics of free Catcher

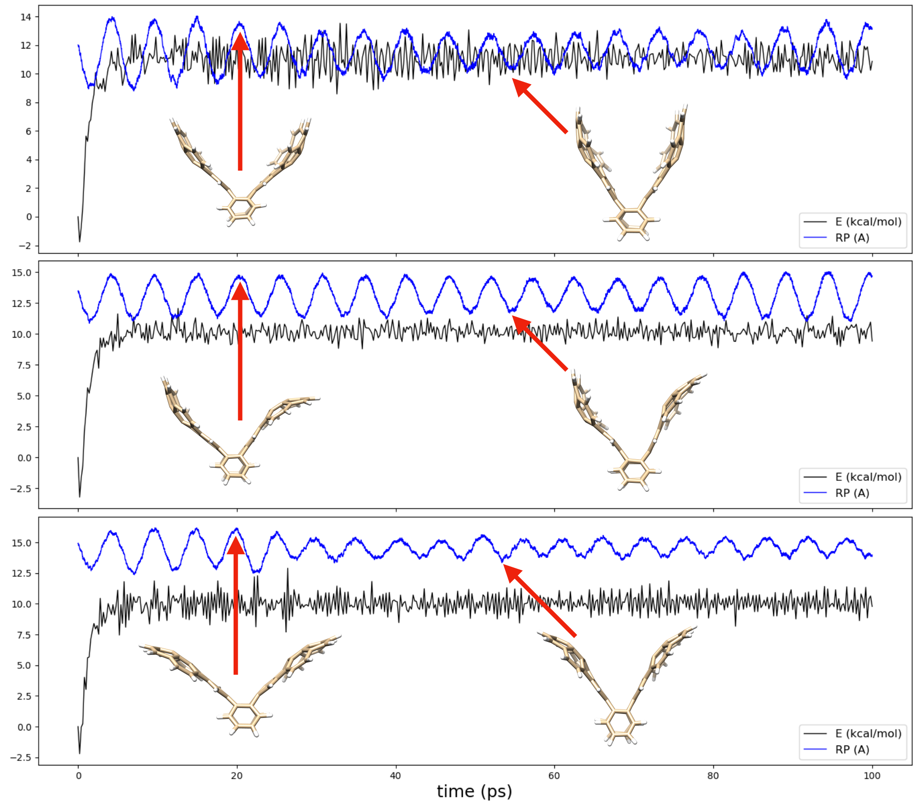

Figure S1: The first 100 ps of the dynamics of each open conformer of the corannulene pincer starting from the minimum in energy.

Effect of Vibrational Scaling Factors

To investigate the possible effects of imprecisions in vibrational frequencies, we recalculated thermodynamic data at the PM6-D3H4X and PM6-D3H+ levels using instead the harmonic mode scaling factor for PM6 (*1*). The resulting effect was marginal, it would however improve Gibbs free energies of aggregation by 0.5 $kcal/mol$. Because scaling factors are average correction factors, which may also deteriorate the quality of vibrational frequencies, we estimate that errors from harmonic modes could be up to 1 $kcal/mol$ in free energies.

Reproducing Conformational Equilibria

In order to build conformational entropies and conformer averaged information one needs to calculate the weights of each conformer in each medium. Technically speaking, gas phase equilibria should be determined according to the gas phase composition and solvation effects introduced according to a different set of weights. We observed however that in our particular case this will not be a determining factor and simply using a single set of conformer weights leads to numerically equivalent results. Furthermore, we tested several solvation models which differ in several aspects. This too lead to numerically identical results. For instance, the gas phase Gibbs free energy for the direct binding of C_{60} different conformer weights for each phase is -12.472 $kcal/mol$. The same quantity using instead the COSMO weights is -12.481 and with the COSMO' weights -12.472 $kcal/mol$.

Conformer Weights for Tetrachloroethane in the Buckycatcher

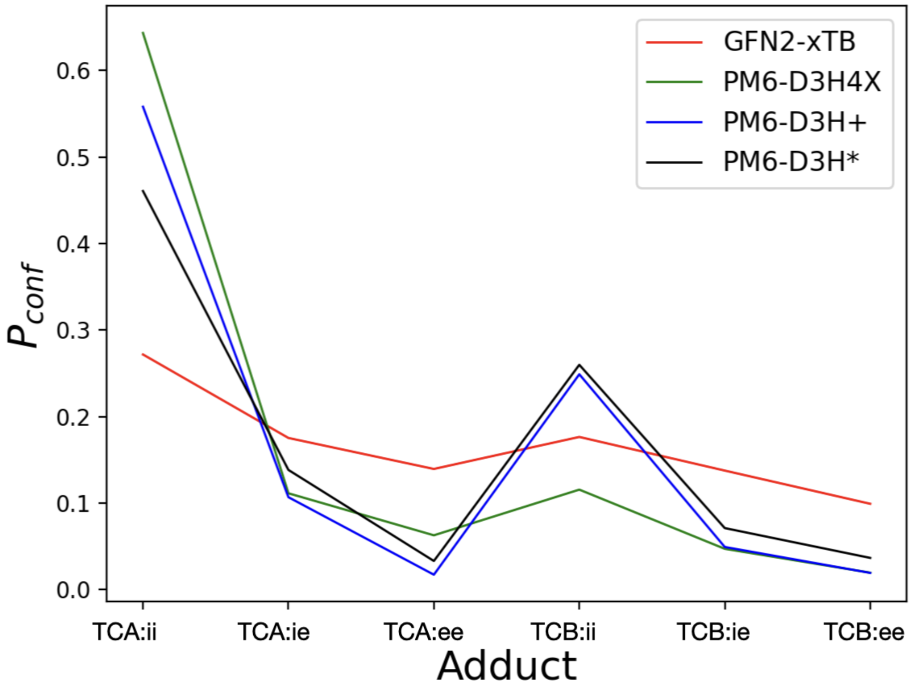

Figure S2: Conformer weights according to the different methods using the ALPB solvation model.

Bibliography

1. R. Johnson, "Computational chemistry comparison and benchmark database http://cccbdb.nist.gov/ doi:10.18434/t47c7z." http://cccbdb.nist.gov/ DOI:10.18434/T47C7Z, 2018. Last checked, 13 July 2021.
